# Fundament and Prerequisites for the Application of an Antifungal TDM Service

**DOI:** 10.1007/s12281-015-0224-3

**Published:** 2015-05-07

**Authors:** Roger J. M. Brüggemann, Rob E. Aarnoutse

**Affiliations:** Department of Pharmacy, Radboud University Medical Center, 864, PO BOX 9101, 6500 HB Nijmegen, The Netherlands; Radboud Institute for Health Sciences, Nijmegen, The Netherlands

**Keywords:** Antifungal agents, Triazoles, Pharmacokinetics, Therapeutic drug monitoring, Isavuconazole, Posaconazole, Voriconazole

## Abstract

Therapeutic drug monitoring (TDM) involves the measurement of plasma or serum drug concentration to adapt dosages to achieve predefined target concentrations that are associated with optimal clinical response while minimizing the chance of encountering toxicity. Many papers in the field of antifungal drugs have focused on the evidence that supports the use of TDM thereby emphasizing the breakpoints or target concentrations in general literature. This review focuses on the process of TDM to inform health care workers on the fundaments and prerequisites that safeguard the good application of TDM. Knowledge on the complete process of TDM including pharmacokinetics (and relevant covariates), pharmacodynamic aspects, trials that are necessary to provide us with evidence, translation of knowledge to other populations and pathogens, and implications for the pre-analytical, analytical, and post-analytical phases (the process of TDM) are discussed in relevant detail. For each individual step, recommendations are made for the readers. We believe this will be a valuable resource and to be of added value to the many papers that focus on relations between exposure and efficacy or toxicity. It will help to achieve greater benefit of TDM.

## Introduction

Currently, three classes of antifungal drugs (i.e., triazoles, echinocandins, and polyenes) are available to the clinician, offering a broader antifungal coverage and/or improved tolerability beyond the initially developed agents in the polyene and triazole classes such as conventional amphotericin B, fluconazole, and itraconazole. Antifungal agents are used either prophylactically [1, 2], empirically, or for targeted therapy [3] for invasive fungal infections. Depending on the strategy chosen, different drugs can be used [4, 5].

Clinical pharmacology encompasses pharmacokinetics and pharmacodynamics. Pharmacokinetics is the study of absorption, distribution, metabolism, and excretion of the drug by the body, whereas pharmacodynamics is the study of the relationship between drug concentrations and drug activity [6]. Pharmacodynamic studies in infectious diseases are related to the activity of the drug against the pathogen. Pharmacodynamics is more than the study of efficacy, it also encompasses the study of drug concentrations in relation to adverse effects that occur. This difference is relevant because the pharmacodynamic parameters associated with efficacy (against the pathogen) may be different from those that define safety (of the patient).

Success of antifungal therapy depends upon the pharmacokinetic and pharmacodynamic properties of the drugs used. There is substantial intra- and interindividual variation in pharmacokinetics and factors causing this variation are not fully known. To truly individualize therapy, all these pharmacokinetic factors must be elucidated in order to adapt drug doses to the individual and achieve optimal antifungal concentrations. Clearly, knowledge on the pharmacodynamics of antifungal drugs is also needed to define the optimal or target concentrations that must be achieved. Therapeutic drug monitoring (TDM) is a strategy to tailor the individuals’ exposure by assessing a patient’s serum or plasma concentration and subsequently adjusting the dosing regimen. TDM integrates pharmacokinetic and pharmacodynamic knowledge at an individual patient level. Over the past decade, much evidence has been published supporting a role for TDM in the class of azole antifungal drugs [7–17].

TDM is increasingly considered a standard of care for antifungal therapy for certain drug-pathogen combinations. Recently, we have reviewed the underlying concepts and indications as well as clinical breakpoints for TDM as a component of antifungal therapy [18, 19]. Also, guidelines such as the upcoming ESCMID guideline on the treatment of Aspergillus infections and the ECIL guidelines will cover the role of TDM (personal communication).

This review provides clinicians the fundaments and prerequisites that safeguard the proper application of TDM. We believe this will be a valuable resource and to be of added value to the many papers focusing on relations between exposure and efficacy or toxicity. For that purpose, several important steps in the process of TDM will be touched upon including the need to have solid information on pharmacokinetics (and relevant covariates), pharmacodynamic aspects, trials that are necessary to provide us with evidence, translation of knowledge to other populations and pathogens, and implications for the pre-analytical, analytical, and post-analytical phases (the process of TDM). Every step has to be controlled to the best optimal extent to use this diagnostic service in its best possible way. The focus will be on the triazole agents voriconazole, posaconazole, and the recently licensed isavuconazole.

## TDM of Antifungal Drugs

For TDM to be useful, it has to fulfill several criteria [20]. Most importantly, there should be large interindividual variability in pharmacokinetics; a good relationship should exist between drug concentrations and effect, a target range should be defined, and no more direct intermediate measure of patient response should be available. Ideally, prospective randomized controlled clinical trials should be performed to validate proposed target values and the clinical utility of TDM.

Many anti-infective drugs meet these criteria and TDM of these drugs is widely applied [21–23]. TDM of antifungals is, therefore, not a new concept. Evidence supporting TDM for the triazoles voriconazole and posaconazole has materialized over the past decade. Evidence of a possible role for TDM of voriconazole first emerged in 2005 and has since continually grown in favor of TDM [24, 25].

For posaconazole, the debate on TDM is still ongoing [7, 17, 26]. With the marketing of the new intravenous formulation and new solid oral tablet of posaconazole, higher exposures are achieved. TDM may still be indicated to prevent overexposure, but currently available data are lacking to substantiate this application of TDM.

For isacuvonazole, there is no evidence available as it has only recently been licensed by the FDA (EMA licensing is still pending).

Despite all research on triazoles in the past decade, additional information with respect to pharmacokinetics and pharmacodynamics of these drugs is needed to fully exploit the concept of TDM for these drugs. In addition, the results of prospective clinical TDM trials are warranted to substantiate the value of TDM. Findings from hematology patients with invasive aspergillosis (where the drugs are most applied) must be translated to other populations (such as ICU, CF) and other pathogens (*Candida* species, *Mucor* species). Only when we have gained all this knowledge can we deploy this diagnostic service to its best possible means.

## Pharmacokinetics of Triazoles

**Fig. 1:**
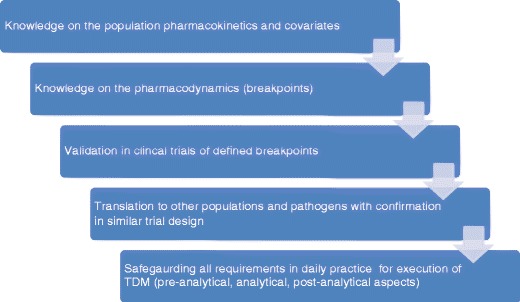
Requirement for a good application of the TDM service

All triazole agents possess different pharmacokinetic profiles. Understanding the differences among agents in this class with regard to absorption, distribution, metabolism, and elimination is essential in order to safely and effectively administer these agents in the complex patient populations at risk for fungal infection. It is also important to appreciate the differences between pharmaceutical formulations of a given drug, since these can be used in one’s advantage (Fig. 1).

Similarly, the pharmacokinetic behavior of antifungals in specific subpopulations must be known. Pharmacokinetic studies typically use healthy volunteers or highly selected patients and focus on the average value of a parameter in the group (i.e., the mean plasma concentration time profile). In such studies, restrictive inclusion/exclusion criteria and controlled designs are often used to increase the internal validity of study findings. However, doing so may obscure the interindividual variability in pharmacokinetics and clinical information on variability that will occur in clinical use. Focusing on a single variable (e.g., renal function) in a traditional pharmacokinetic study also makes it difficult to study interactions among variables.

In practice, however, certain demographic, pathophysiologic, and therapeutic features, like body weight, excretory and metabolic functions, and the administration of other therapies, can regularly alter dose-concentration relationships. By performing population pharmacokinetics, one is able to identify the measurable pathophysiologic factors that cause changes in the dose-concentration relationship and the extent of these changes.

With regard to azole therapy, insufficient population pharmacokinetic data are available for various subpopulations of patients. Specifically, there are limited data in intensive care unit patients, cystic fibrosis patients for both voriconazole and posaconazole. Voriconazole pharmacokinetic data are well documented in pediatric patients, but data on posaconazole in this population are lacking. Fortunately, a vast amount of pharmacokinetic data for posaconazole and voriconazole in adult hematology patients exist [9, 27–33]. Data on isavuconazole are not yet extensively available.

These data are urgently needed to build population pharmacokinetic models and to reveal factors associated with variation in specific populations that can assist in decision-making. Once population parameters are known, interpretations for an individual patient can be made to help define optimal dosing regimens needed to achieve predefined target. Furthermore, it helps in interpretation of results returned from the pharmaceutical laboratory. This aspect is addressed with antifungal therapeutic drug monitoring.

## Pharmacodynamics of Triazoles: Clinical Breakpoints

Patients achieving high concentrations of azole drugs may show improved response to therapy but may also be at higher risk for toxicity. Conversely, patients achieving lower concentrations may have reduced therapeutic response but subsequently be at lower risk for adverse events. The safe and effective target concentrations (or “therapeutic window”) for most antifungals have not been definitely established. The questions remain: how can one identify those breakpoints that best fit the population at risk or having invasive fungal disease?

Many large trials have not addressed the relation between dose and effect [34–38]. Ideally, studies, particularly those involving the azoles should include a (post hoc) pharmacokinetic and pharmacodynamic analysis to identify a relation between concentration and effect or toxicity and help define initial estimates for target concentrations because dose does not correlate with plasma concentrations for voriconazole and posaconazole. Without these trials being, it is almost impossible to identify clinical breakpoints.

There is an important need for regulatory agencies to require such analyses from the pharmaceutical industry thereby providing more insight into possible clinical breakpoints. To date, with a few exceptions, a gap exists in that few pharmaceutical industry research initiatives are designed to conduct such research [7, 28, 39]. Commercially, the need to conduct TDM carries a negative perception because it adds to the cost of using the drug. Conversely, clinically, TDM is viewed positively because it is a tool used to further improve individual therapy.

## The Need for TDM Trials for Antifungal Drugs

More studies are warranted to assess the value of TDM of antifungal drugs in both the setting of primary or secondary prophylaxis and primary or salvage treatment. A voriconazole TDM trial in the setting of invasive aspergillosis is ongoing in the Netherlands, but voriconazole TDM also needs to be validated in the setting of prophylaxis.

With the advent of newer dosage forms presents the ideal opportunity to start a posaconazole international TDM trial investigating and validating the clinical breakpoints of posaconazole for prophylaxis and therapy. An international trial rather than one limited to one country would be needed because large numbers of patients will be required to make valid statements regarding prophylaxis cutoff values as well as therapeutic targets. When initiating a TDM trial to validate clinical breakpoints, it is crucial not to start to early as preliminary breakpoints may change with emerging evidence. Such trials should also not be delayed, because eventually all physicians may become acquainted with TDM of posaconazole even when it is not warranted. When such familiarity is reached, performing a TDM study may be considered unethical.

## Translation of TDM Knowledge into Clinical Practice: How to Translate Findings from TDM in One Host with One Bug, to Another Host with Another Bug?

Once the results from the TDM trial in, for instance, hematology patients with invasive aspergillosis are obtained, how do such data translate to other populations like intensive care patients with invasive aspergillosis or to hematology patients with an invasive candidiasis or a mucormycosis? Do the same criteria and breakpoints still apply?

These questions are difficult to answer. Knowing the pharmacokinetics in the target population by measuring plasma concentrations will circumvent problems such as malabsorption or low plasma concentrations in patients with increased volumes of distribution. Measuring plasma concentrations will enable making preventive measures to avoid subtherapeutic exposure or unnecessary toxicity. Performing TDM will help address pharmacokinetic differences between different patient populations.

Differences in pharmacodynamics are more difficult to address. Ideally, the same study is performed in other populations and patients with other causative microorganisms. In practice, the same breakpoints can be used for more susceptible species such as *Candida albicans* and perhaps for non-neutropenic patients, in whom the immune system and antifungal therapy will target the invasive mycosis. Yet, this will not provide the ultimate scientific evidence, and other challenges may exist including how to deal with polymicrobial infections, microorganisms with reduced susceptibility, and patients with combination therapy.

As there are two pharmacodynamic endpoints, one for efficacy against the pathogen and one for human toxicity, attention must be paid also to the latter. With regard to toxicity, subjective translations from one population to another are more straightforward.

Even if the ideal TDM study for every condition has been conducted, it should be noted that the notion of a therapeutic range is more a probabilistic concept than an absolute entity. It represents a range of drug concentrations within which the probability of a desired clinical response is relatively high and the probability of unacceptable toxicity is relatively low. In addition, extrapolating findings based on population research to the individual is challenging. Some patients respond effectively below the therapeutic range, whereas others need concentrations above it. Similarly, some patients experience toxic reactions within the therapeutic range. Common sense and sound clinical judgment must remain the cornerstone of a patient’s treatment, and TDM is a diagnostic tool that may help explain the clinical course of a patient.

## TDM as a Process

**Fig. 2:**
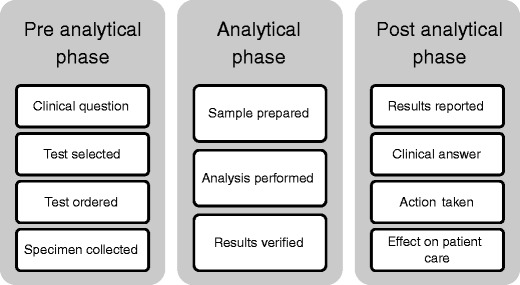
TDM as a process: “the total testing process” with 3 components and 11 steps (adapted and modified from [48]

Performing TDM must be considered as a complex process in which all phases must be known to the users involved (Fig. 2). Specifically, TDM must be considered a multidisciplinary process. All professionals involved, from nursing staff and physicians to analytical staff, pharmacologist, and pharmacist, all carry specific responsibilities. Each and every one must be aware of the interrelated steps that need to be most advantageous setup to safeguard the proper use of TDM.

The clinician must use results from TDM to answer the clinical question they raised when the measurement was ordered. Clinical pharmacists/pharmacologists can provide an interpretation of plasma drug concentration and make recommendations on how to optimize patient dosages. The clinical microbiologist can provide input with regard to susceptibility of cultured species. All three professionals must be acquainted with the population for which the test is ordered and have sufficient knowledge on the individual’s background. Only when a clinician works closely with the clinical pharmacist and microbiologist, the multidisciplinary approach of TDM of antifungals can be beneficial.

Once established, adherence to guidelines on TDM is crucial in obtaining the optimal result for the individual. TDM emphasizes that pharmacokinetic-guided dose recommendations should not be viewed simply as a numerical value for plasma drug concentrations. Training and teaching of everybody involved is thus an essential requirement and must be done not only when setting up this service but moreover also during the wide application of TDM.

The process of TDM involves basically three phases. These phases involve a pre-analytical phase, an analytical phase, and a post-analytical phase. Each individual phase is subdivided into substages as illustrated in Fig. 2.

### The Pre-analytical Phase

How early can we draw a TDM sample: The therapeutic range for a drug is based on steady-state plasma concentrations. Concentrations drawn too soon after a dosage regimen has been started or changed and may provide misleading information. In the case of voriconazole, a first sample can be drawn on day 3 of therapy. But for posaconazole, steady-state conditions are only reached by days 7–10. Earlier assessment of plasma concentrations will facilitate making timely interventions and bring decisions to a prior time point. An algorithm has been suggested to determine samples early after the start of therapy (day 3) and targeting a lower concentration that will eventually result in a final target concentration under steady-state conditions [7]. More importantly, software is now available for dose predictions.

### At What Time Point Should We Draw the Sample

The sampling time will influence outcomes from TDM specifically in drugs with a very short half-life such as voriconazole. In previous studies, TDM sampling for voriconazole have often been performed randomly, which makes interpretation of the results difficult [24, 25]. The ultimate sampling scheme would encompass a certain amount of sampling moments that would best predict total exposure (area under the concentration versus time curve), but this method is considered unfavorable for many reasons such as patient burden, required nursing time, and complex logistics. In routine practice, the most common sampling moment is just prior to the next dose (trough concentrations or *C*_min_). Using a single sample will reduce costs and more importantly will reduce patients’ burden due to frequent sampling. Since trough concentrations correlate with exposure, this provides a measure that in clinical practice can be easily introduced. Posaconazole has a very long terminal half-life. Therefore, peak and trough concentrations differ minimally, especially with frequent dosing (i.e., three or four times daily). Sampling at trough concentrations is therefore not necessary but may only be preferred from a practical point of view.

### The Analytical Phase

The basis for clinical pharmacokinetic research and TDM is the availability of an accurate, precise, sensitive, and selective analytical method for the quantitative determination of azole antifungal drugs and their metabolites in plasma/serum, with additional methods for other matrices such as plasma, urine, cerebrospinal fluid, and tissue if possible [40–42]. In addition, analytical methods for measurement of free, protein unbound antifungal drugs will provide insight into the amount of protein binding under certain circumstances and enable the measurement of active drug, since only free drug can exert a pharmacological effect. Assays have to be validated according to the current requirements for validation of bioanalytical assays [34]. To help identify sources of errors and to further improve analytical methods, participation in an ongoing proficiency testing program is recommended [35]. Results from a recent 5-year analysis of an international proficiency testing program show that 1 out of 5 analyses lie outside the predefined range of 80 to 120 % of the weighed-in concentrations indicating an improper report on the concentration [36]. The performing laboratory is the main determinant of inaccuracy, suggesting that internal quality assurance is pivotal in preventing inaccuracies, irrespective of the antifungal drug measured, concentration, and analytical equipment. There is a strong need for an ongoing proficiency testing program to further improve the analytical methods for routine patient management.

### Post-analytical Phase

Interpretation of the results can be done preferably by a clinical pharmacist/pharmacologist who is aware of all critical information as only then can a solid advice be given. This information involves, among others, knowledge on sampling time (directly after loading dosage or during a steady state) and knowledge on the population (neutropenic, CF, ICU), patient details (disease, phase of treatment, mucositis, drug interactions), therapeutic range for the specific population, and pathogen combination. Most importantly, the professional should maintain knowledge on developments and relevant discussion in literature. The advice from the clinical pharmacologist must be brought into practice by the clinician and the microbiologist must be informed (this can be done in a wide variety of ways). Assuring adequate effect on the patient is the clinician’s responsibility. Not achieving the desired result must prompt repetition of the previous cycle of steps.

Software that helps to define optimal dosing strategies are currently available or under development (i.e., MwPharm, DoseMe, BestDose). Using this software allows early clinical decision-making as one does not have to wait until steady state has been reached. The models defined will assist in making predictions under steady-state conditions. In addition, with a single or preferably multiple measurements that serve as prior input, an area under the concentration time curve can be calculated. All prior inputs will also serve for model refinement (for the population) and predictions for the individual done at a later stage. Another advantage is that non-trough concentrations can be used as input to predict extrapolated trough concentrations or AUC under the condition that administration and sampling time are recorded adequately.

Lastly, the frequency of sampling remains subject to ongoing debate. One single sample is not considered TDM and will not provide the necessary input on a patient’s intraindividual pharmacokinetic variability. When TDM is commenced, this means multiple interventions over time are sometimes necessary to achieve target concentrations to remain within the therapeutic range. As mentioned previously, there is substantial intraindividual variation in triazole drug concentrations over time. Due to this high intrasubject variability, an optimal sampling frequency has to be determined to timely adjust dose in order to achieve concentrations within the target range. A good starting point would be to perform TDM once or twice a week shortly after initiation of therapy. This can be reduced to once every 2 weeks when the patient is clinically improving or even less frequent when the patient is no longer in the hospital. Changes in clinical condition or when interacting drugs are introduced may prompt for more frequent sampling even when therapy has been given already for a longer period of time.

## Conclusion

### TDM of Antifungal Drugs in the Future

The issues discussed provide opportunities for future research in the next couple of years. But what about a long-term forecast? Where will we stand in 10 years? Will all be the same or does the current research help us to induce a change in the way TDM is used? Again, several steps are crucial: (1) industry must fulfill a prominent role in gathering data on breakpoints, (2) optimal initial dosing regimens must be known for the specific subpopulation, (3) sample assessment must be brought to the absolute earliest time after treatment initiation, and finally (4) new sampling techniques must be developed to provide the basis for continuous monitoring. All steps are detailed in the next paragraph.

### The Role of the Pharmaceutical Industry

At this time point, TDM is warranted since the current dosing regimens do not fit the individual. Some antifungals have been licensed at a dose that subsequently has been changed. For example, both the adult and pediatric dosing guidelines of voriconazole have changed since market introduction. This indicates that some antifungals were marketed lacking sufficient data to identify the optimal dosages in specific cohorts of patients. Unfortunately, antimicrobial research has diminished for a variety of reasons [37]. Investigator-initiated research lack the scale needed to generate sufficient data. Therefore, the pharmaceutical industry should perform large clinical trials to further elucidate pharmacokinetic and pharmacodynamic behavior of drugs in special patient populations. Large clinical trials need to incorporate pharmacokinetic research instead of only investigating the effect of dose on outcome [38, 43–45]. It is unclear from these studies if failure to respond to therapy originates from underexposure or toxic exposure since plasma concentrations are unknown. This research should be performed before or very short after market authorization.

### Delay

At the moment, TDM can be considered a diagnostic tool. Diagnostics may be useful to detect a change in a clinical condition such as an infection or in the case of TDM a sub- or supra-therapeutic exposure. Unfortunately, diagnostics always follow a change in clinical condition that necessitates frequent monitoring. The inherent downside of TDM is the possible delay in first-time assessment as well as follow-up assessments. This may be due to pharmacokinetic aspects (i.e., steady state not reached), cost aspects, as well the availability of in-house testing facilities. A delay in the possibility to timely determinate too low or too high exposure may have clinical consequences. It has been repeatedly shown that delay in the initiation of antifungal therapy and inadequate exposure are independently associated with increased hospital mortality.

### Upfront Adequate Dosing

We must orchestrate our knowledge derived from population pharmacokinetic models to seek for a probabilistic (or stochastic) model to upfront give the patient the optimal dose based on specific patient features (identified covariates). This implies that we would switch from TDM as a concentration-guided dosing tool to an approach where the drug concentration could be used as a validation set of an individualized dosing advice and thereby bringing it to earlier time point with subsequent reduction in host risk. Also, whenever possible, an attempt should be made to integrate pharmacodynamic parameters such as a pathogen’s MIC. This approach, with a tailored dose followed by validation of target concentrations, will be performed very soon (i.e., 2 or 3 days) after the start of therapy. Inherent to whatever new approach is chosen, we may not be able to identify all factors that cause changes in PK/PD. In other words, a residual error will always be present and unforeseen changes in the clinical situation will prompt for new assessments of concentrations. But I strongly believe that we should put effort into bringing this diagnostic tool to an earlier time point after initiation of therapy with a tailored dose.

### New Sampling Techniques

Unfortunately, many times sampling refrains to the setting of clinical monitoring and the patients are lost to follow-up after leaving the hospital. Also, monitoring in a home-based setting might be hindered by the difficulty of obtaining easy venous access, patient discomfort, technical issues such as instability of the drug sample, and logistical problems. All these may be solved by applying the new sampling technique termed dried blood spot (DBS) sampling. In DBS sampling, blood is obtained via a finger prick with an automatic lancet. With clear instructions and after adequate training, patients administer this finger prick themselves. The sample is then dried and sent by regular post to the laboratory for analysis. The technique is well established for applications such as neonatal screening for inborn diseases, but has recently experienced a surge of interest in the context of therapeutic drug monitoring (TDM). This technique is very suitable not only for continuous monitoring at home but also for the purpose of pharmacokinetic research outside the hospital. Furthermore, it allows clinicians and other health care workers who do not have access to onsite facilities to send the samples by regular mail to a reference laboratory. Currently, the Radboudumc has such an assay available supporting this approach. Other similar initiatives have been reported in literature [46, 47].

In summary, clinical pharmacological research of antifungal drugs remains highly relevant for the individualization of treatment of patients. The challenges of TDM of antifungal drugs in a wide cohort of patients provide an interesting opportunity and challenge for future research to further optimize the individual’s treatment. Knowledge on each individual step in this process, and the optimization of these steps, will allow for optimal application of this diagnostic service.
